# Nutritional Status in Intensive Care Unit: A Meta-Analysis and Systematic Review

**DOI:** 10.31661/gmj.v9i0.1678

**Published:** 2020-10-08

**Authors:** Mohammed Ibrahim Mohialdeen Gubari, Mohammad Javad Hosseinzadeh-Attar, Mostafa Hosseini, Fadhil Ahmed Mohialdeen, Haval Othman, Khalid Anwar Hama-ghareeb, Abdolreza Norouzy

**Affiliations:** ^1^Department of Clinical Nutrition, School of Nutritional Sciences and Dietetic, Tehran University of Medical Sciences, Tehran, Iran; ^2^Centre of Research Excellence in Translating, Nutritional Science to Good Health, The University of Adelaide, Adelaide, Australia; ^3^Department of Epidemiology and Biostatistics, School of Public Health, Tehran University of Medical Sciences, Tehran, Iran; ^4^Community Health Department, Technical College of Health, Sulaimani Polytechnic University, Sulaimani, Iraq; ^5^General Shar Teaching Hospital, ICU Unit, Sulaimani, Iraq; ^6^General Director of Health, Research Center Department, Sulaimani, Iraq

**Keywords:** Nutritional Status, Intensive Care Unit, Systematic Review, Meta-Analysis

## Abstract

It is important to consider the nutritional status of patients in the intensive care unit (ICU) since it is a key element in the ability to overcome and survive critical illnesses and clinical outcomes. The aim of the present study was to provide a meta-analysis and systematic overview in determining the nutritional status of patients in ICU by examining other studies. All studies published during 2015-2019 on nutritional status in ICU were retrieved from Medline (via PubMed), Embase, Scopus, and Web of Science databases. Finally, 23 articles were included in the meta-analysis. Results obtained from these studies showed that the nutritional status of patients in ICU was inappropriate (the pooled proportion of malnutrition was 0.51 in the type of study stratified), in which many patients in this unit had different degrees of malnutrition (moderate-mild malnourished and severe malnutrition is 0.46 and 20%, respectively). According to the results of this study, the nutritional status of patients in ICU was unsatisfactory; hence, it is necessary to consider the nutritional status along with other therapeutic measures at the beginning of the patient’s admission.

## Introduction

The intensive care unit (ICU) is a specialized ward at the hospital, in which patients with severe problems are admitted and undergo constant care and close supervision [1]. Most patients in ICU are unable to maintain a healthy diet due to their life-threatening and sometimes unconscious conditions [2]; therefore, paying attention to the nutritional status of patients in these units is very important and is considered as one of the main factors in these wards [3]. In ICU, the nutritional status is a key factor in the ability to overcome critical diseases and to improve clinical outcomes [4,5]. Nutrition and disease are closely related [6]. The reduction of nutrient intake, along with the increase in body needs and/or the use of modified nutrients, brings about the need to maintain homeostasis in ICU patients. On the other hand, these patients tend to have metabolic stress following a critical condition, in which they develop systemic inflammatory responses [7]. Consequently, metabolism increases, and if adequate calories and protein are not provided for a healthy metabolism, it increases catabolism, reduces fat storage, and decreases muscle mass [8]. These conditions lead to protein-energy malnutrition (PEM), which is a major problem of hypercatabolic patients with severe conditions in the ICU [6,8]. Studies have shown that malnutrition in ICU patients is more compared to other patients [9,10]. In a study by Verghese *et al*., it was shown that all the studied patients admitted to the ICU had different levels of malnutrition [11]. Singh *et al*. revealed that the calorie and protein intakes of ICU patients were lower than the recommended level, and this is associated with a high mortality rate [12]. Many of the problems associated with PEM of ICU patients include the increase in hospital infections due to reduced immune function, delayed wound healing due to decreased tissue repair, delay in mechanical ventilation device isolation of patients due to changes in vital functions of the body and, depression and mental disorders [13]. One of the many factors identified in the etiology of malnutrition is the decreased food intake during hospitalization. Adequate daily intake is an essential factor in the treatment of malnutrition [14]; therefore, nutritional status has an impact on the ability to overcome critical conditions and clinical outcomes, especially in ICU patients. Inadequate food intake in these patients, in addition to nutritional deficiencies, can cause deterioration of health conditions and accelerate the onset of many disorders. The present study was conducted to determine the nutritional status of patients admitted to ICU.

## Materials and Methods

The systematic review and meta-analysis were performed according to the meta-analysis of observational studies in epidemiology (MOOSE) guidelines [15].

###  Search Strategy

 We used four databases: Medline (via PubMed), Embase, Web of Science, and Scopus in this study. The search was restricted to the years 2015 to 2019 because the nutritional status and prevalence of malnutrition in recent years was the focus of the present study. Keywords related to nutritional status in combination with words related to ICUs were used for search.

###  Inclusion and Exclusion Criteria

In the present study, we included studies that were published between 1st^st^January 2014 to 16^th^ August 2019, were cohort, case-control, or cross-sectional studies, involved ICU unit type, had patient’s referral date after 31st^st^December 2013, and involved nutrition/malnutrition status. Also, old literature, pediatric, in which the patient’s referral date was before 31st^st^December 2013 studies were excluded from the systematic review.

###  Data Extraction

 After applying the inclusion and exclusion criteria for eligible studies, items such as first author name, sample size, number of malnutrition cases, method of obtained nutrition status, and findings were independently extracted by two reviewers after carefully reviewing the articles.

###  Quality Assessment 

The quality of studies included in the meta-analysis was assessed using the Newcastle-Ottawa Scale (NOS) [16]. According to the NOS, studies scoring seven or more were regarded as having a low risk of bias; 4–6 a modest risk of bias; and studies <3 were considered to be at substantial risk of bias [17].

###  Statistical Analysis

The proportion of the number of malnutrition cases to the total number of patients was analyzed using the metafor package in R software version 3.6.1 (https://www.r-project.org/) [18]. To assess the homogeneity between the studies, the Cochran’s heterogeneity (Q) and I2 statistics were used. Based on these statistics, the fixed effect and random-effect models were applied to obtain the pooled proportion of the number of malnutrition cases [19]. Also, to assess publication bias, Egger’s regression test for asymmetry studies was used [20,21].

 We used two strata (severe and mild-moderate) in the present study since all studies did not indicate all malnutrition status (severe, moderate, and mild). Therefore, the stratified analysis was used to identify the burden of overall malnutrition status. Also, subgroup analysis performed for the type of studies include cross-sectional, case-control, and cohort studies as well as developed and developing countries for the proportion of patients with malnutrition regardless of malnutrition status.

## Results

###  Study Selection

**Figure 1:**
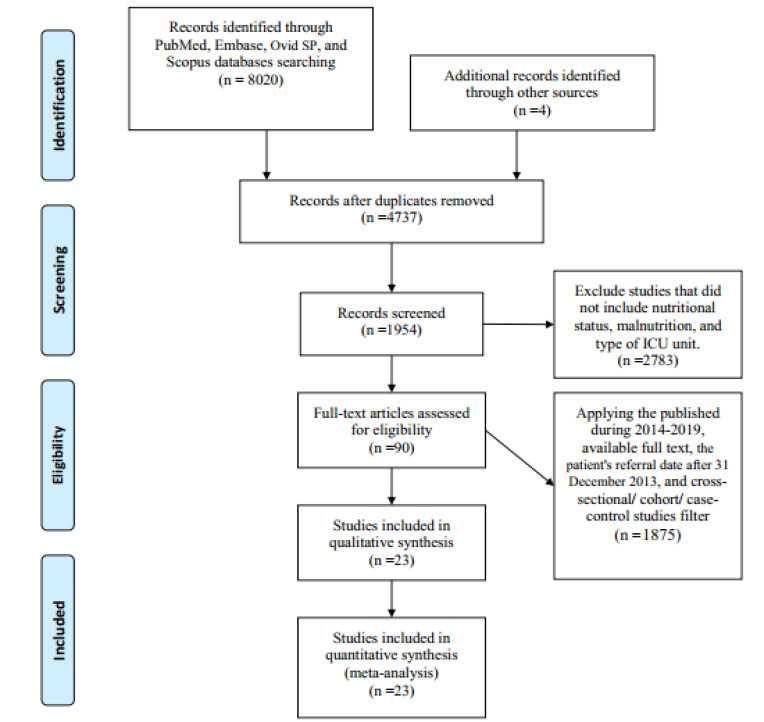
Flowchart of study

After a search in databases, we detected 8024 records (PubMed: 1571, Embase: 3126, Web of Science: 460, Scopus: 2863, and other sources: 4). Of these studies, 3287 were duplicates, 2873 did not include nutritional status, malnutrition, as well as the type of ICU unit. Then, 1875 records were removed after applying the filters (published during 2014-2019, the patient’s referral date after 31st^st^December 2013, and cross-sectional/ cohort/ case-control studies). After the screening of titles, abstracts, and full-text screening, 23 records [22-44] were included for systematic review and meta-analysis (Figure-1).

###  Characteristics of Studies

 From a total of 30942 subjects included in the 23 studies, 6845 subjects had malnutrition. The mean age of the subjects was 59.63 years. In all included studies, five studies were cross-sectional, two studies were case-control, and 16 studies were cohorts. Also, from these studies, only 13 studies indicated malnutrition status (the three malnutrition status in severe, moderate, and mild). Further details are shown in Table-1.

###  Overall Publication Bias

**Figure 2:**
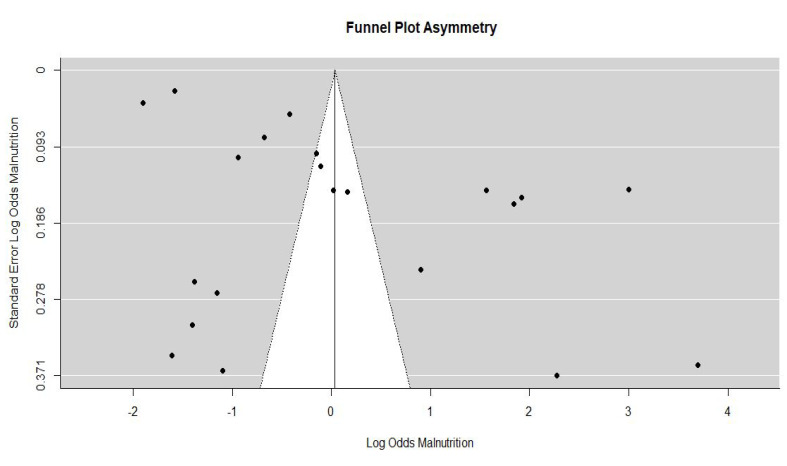
Funnel plot asymmetry for publication bias in 23 studies.

 Based on the funnel plot, Egger’s, and rank regression test, there was a significant publication bias between studies. The P-value of Egger’s regression test was 0.004. The funnel plot is presented in Figure-2.

###  Stratified Malnutrition Status

 The present meta-analysis consists of three stratified malnutrition status, including severe, moderate, and mild. Therefore, since all the studies did not include all three status, we combined the moderate and mild conditions and compared them with the severe condition.

**Figure 3:**
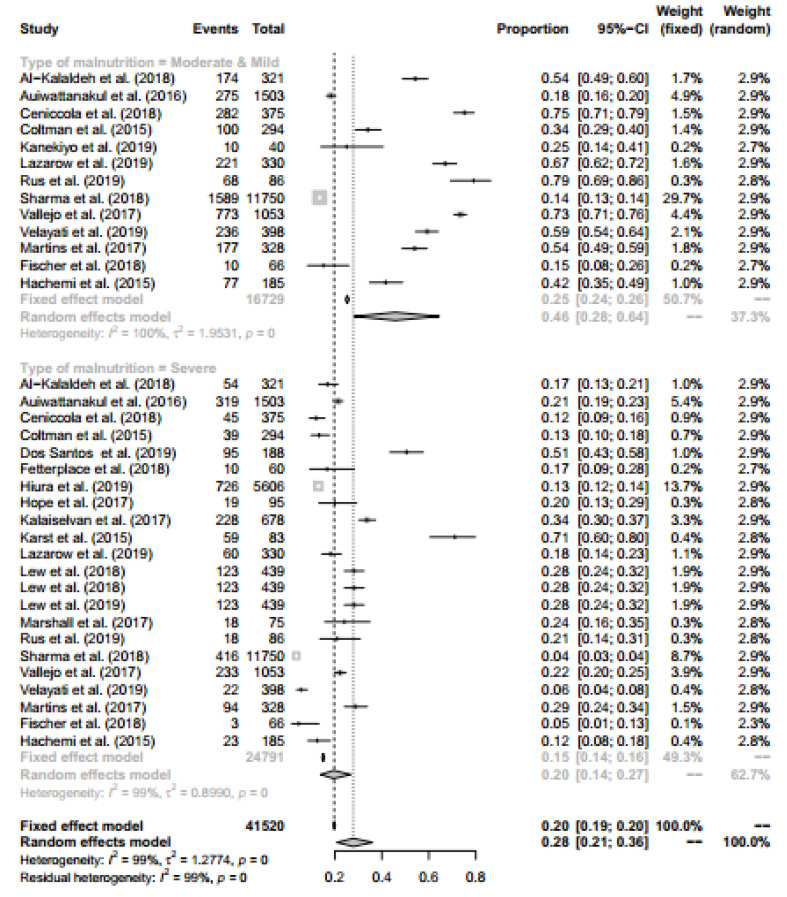
Forest plot for stratified malnutrition status.

 The results of this section show that the proportion of people who are mild-moderate malnourished and severe malnutrition is 0.46 (with a 95% confidence interval [CI] 0.28 – 0.64) and 0.20 (with a 95% CI 0.14 – 0.27), respectively. Since heterogeneity was higher than =98% (P<0.01), a random effect model was used to construct the combined confidence interval. The Forest plot for stratified malnutrition status is presented in Figure-3.

###  Subgroup Analysis

**Figure 4:**
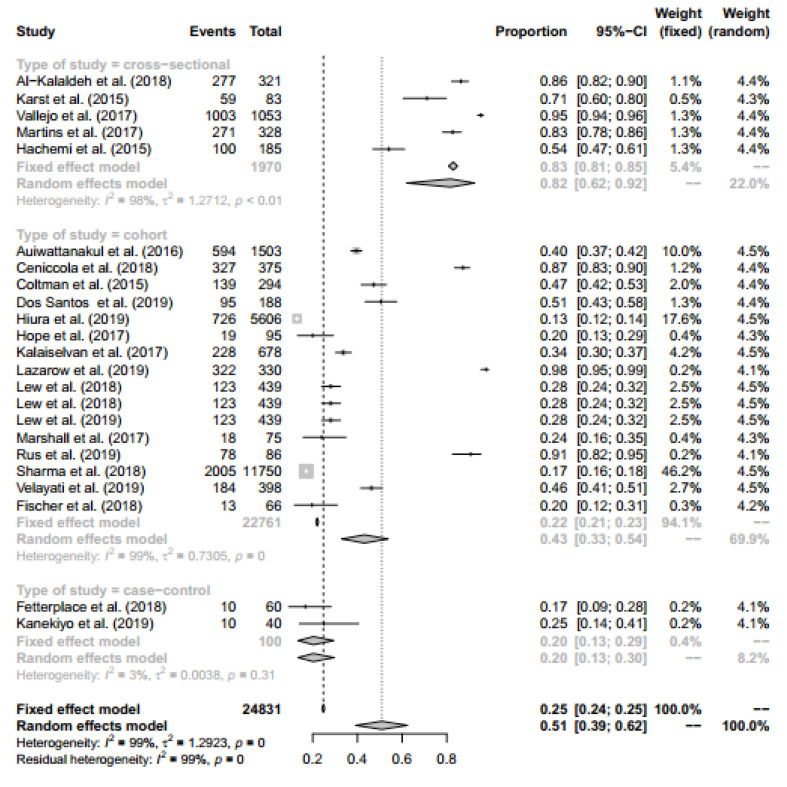
Forest plot for cross-sectional, case-control, and cohort studies subgroup analysis.

**Figure 5:**
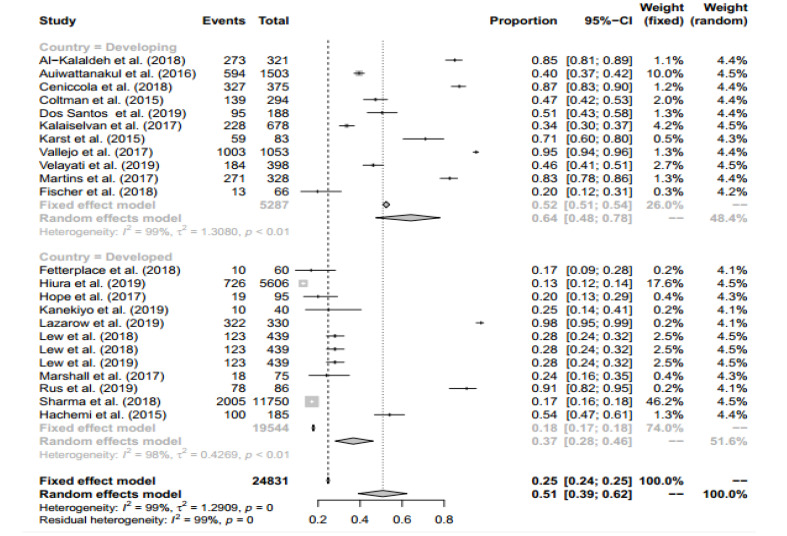
Forest plot for developed and developing countries subgroup analysis.

 Subgroup analysis was performed for all included study types (cross-sectional, case-control, and cohort studies) and countries development (developed and developing). Therefore, the proportion of people who are malnourished in cross-sectional/case-control/ cohort studies and developed/developing countries are 0.82 (95% CI: 0.62 – 0.92) / 0.2 (95% CI: 0.13 – 0.30) / 0.43 (95% CI: 0.33 – 0.54) and 0.37 (95% CI: 0.28 – 0.46) / 0.64 (95% CI: 0.48 – 0.78), respectively. Finally, the pooled proportion in the two subgroups analysis was 0.51 (95% CI: 0.39 –0.62). Forest plot for subgroup analysis is presented in Figures-4 and 5.

###  Evaluated Studies

 Based on the three categories of NOS, the total score for one study is 8; for two studies is 7, for two studies is 6, for five studies is 5, for five studies is 4, for six studies is 3, and for two studies is 2. Assessments of studies are shown in Table-2.

## Discussion

These studies have shown that the nutritional status of patients in ICU is inappropriate with a high percentage of different degrees of malnutrition (the pooled proportion was 51%). Also, severe malnutrition in this unit is 20%, and for developing countries is 64%. Malnutrition is a serious problem among many ICU patients [8]. Studies have shown that not paying attention to the nutritional needs of ICU patients can lead to deterioration of the disease, increased length of the disease, ventilator dependence, and high cost [34,35,45,46].

Studies also indicate that disruption in the provision of nutritional needs of ICU patients leads to a higher calorie deficit during critical periods of the disease. Some factors which can cause inadequate nutrition in patients include nutritional disruption for diagnostic procedures, nutrition discontinuation in managing the remaining gastric ulcer, lack of nutritional requirements, and delayed nutritional support [2,9]. In modern medicine, the concept of “nutrition therapy” is a substitute for supportive nutrition, which plays a vital role in the nursing care of ICU patients [3]. Relatively, specific measures that have to be taken include periodic visits by a nutritionist and implementation of nutritional guidelines for ICU patients. Studies have shown that nutritional counseling, along with diverse strategies of a nutritional support team at the hospital, especially ICU, has led to a reduction in the prevalence of malnutrition [47,48]. The presence of experts and nutritional support team can significantly improve the performance of ICU staff by providing adequate nutritional support [49]. In a study performed by Park *et al*., the presence of a nutritional support team had a positive and significant effect on the nutritional and clinical outcomes of ICU patients [48]. Evidence suggests that using these guidelines and nutritional protocols can help increase nutritional adequacy and prevent complications arising from inappropriate nutrition in ICU patients [50-52]. ICU patients are a heterogeneous group, and in order to meet their nutritional needs, a single approach cannot be used for each patient. The medical diagnosis of the different stages of the disease (early, post-recovery, stabilized, long-term residence) and any other complications should be taken into account simultaneously [2]. Nevertheless, the protocols provided by the European Society for Clinical Nutrition and Metabolism (ESPEN) present a set of nutrient recommendations in most clinical cases of the ICU [53]. Some of the advantages of using ESPEN protocols include timely and correct identification of high-risk patients, nutritional evaluation of ICU patients, determination of energy needs for each patient, and selecting appropriate methods to provide nutritional support based on the patients’ clinical conditions [2].

## Conclusion

 The results of this study revealed that the nutritional status of patients in the ICU is inappropriate, and most ICU patients are facing varying degrees of malnutrition. Malnutrition was associated with unfavorable clinical outcomes, such as increased length of stay in ICU, the duration of mechanical ventilation, and mortality rate. Therefore, it is necessary to accurately analyze the nutritional status of patients at the beginning and during their admission and to implement nutritional guidelines developed for the ICU by a professional nutritional support team, including nutritionists, physicians, and nurses.

## Conflict of Interest

 The authors declare no conflict of interest.

**Table 1:** Characteristics of the Included Studies.

**Author**	**Country**	**Type of study**	**Sample size**	**Mean age**	**Male gender**	**Sampling method**	**Type of feeding**	**Malnutrition criteria**	**Type of malnutrition**	**Follow-up**
Al-Kalaldeh *et al*. (2018)	Jordan	Cross-sectional	321	60.03	211	Convenience	Tube feeding enteral nutrition	MUST and Phase Angle	MUST (low risk : 125, medium risk:65, high risk:38) Phase angle (severe:54, moderate:79, mild: 95)	NA
Auiwattanakul *et al*. (2016)	Thailand	Cohort	1503	65	860	Convenience	Oral: 1375 Tube feed: 53 IV: 17 Combined: 43 none: 15	NRS-2002 score	Severe:319 Moderate:130 Mild:145	28 days
Ceniccola *et al*. (2018)	Brazil	Cohort	375	Non-malnutrition: 49.8 Malnutrition: 57.61 Severe malnutrition: 59.85	Non-malnutrition: 151 Malnutrition: 60 Severe malnutrition: 31	Convenience	Enteral nutrition	AND-ASPEN criteria	Not malnutrition: 229 Severe: 45 Moderate: 53	Until discharge or death
Coltman *et al*. (2015)	NA	Cohort	294	59	Total: 146 Malnutrition: 50	Convenience	Oral	SGA and NUTRIC	Severe:39 Moderate-mild:100	3 month
Dos Santos *et al*. (2019)	Brazil	Cohort	188	48.5	134	Convenience	NA	BMI and AC	Severe	12 month
Fetterplace *et al*. (2018)	Australia	Case-control	60	56	44	Random	Parenteral nutrition Enteral nutrition Tube feeding	SGA	Severe	15 days
Hiura *et al*. (2019)	America	Cohort	5606	NA	3029	Convenience	Enteral nutrition		Severe	12 month
Hope *et al*. (2017)	America	Cohort	95	57.1	51	Convenience	NA	Weight loss	Severe	10 month
Kalaiselvan *et al*. (2017)	India	Cohort	678	55.7	458	Convenience	NA	mNUTRIC score ≥ 5	Severe	24 month
Kanekiyo *et al*. (2019)	Japan	Case-control	40	63.5	32	Random	Enteral nutrition oral	SGA	Well-nourished: 30 Mild-moderate malnutrition: 10 Severe malnutrition : 0	3 day
Karst *et al*. (2015)	Brazil	Cross-sectional	83	68.7	52	Convenience	NA	SGA and APMT	Severe	4 month
Lazarow *et al*. (2019)	America	Cohort	330	59	192	Consecutive	NA	MST	Low malnutrition: 261 High malnutrition: 60	24 month
Lew *et al*. (2018)	Singapore	Cohort	439	61.4	259	Consecutive	NA	SGA and mNUTRIC	Severe	00014 month
Lew *et al*. (2018)	Singapore	Cohort	439	61.6	257	Consecutive	NA	SGA	Severe	14 month
Lew *et al*. (2019)	Singapore	Cohort	439	61.6	259	Consecutive	NA	SGA	Severe	14 month
Marshall *et al*. (2017)	Australia Canada	Cohort	75	59.3	43	NA	Enteral nutrition oral	MST	Severe	15 month
Rus *et al*. (2019)	Romania	Cohort	86	61.4	54	Consecutive	NA	CONUT score	Severe:18 Moderate-mild:60	30 days
Sharma *et al*. (2018)	New Zealand	Cohort	11750	NA	6276	NA	Oral	MUST	Severe:416 Moderate: 270 Mild:1319	12 month
Vallejo *et al*. (2017)	Argentina Brazil Chile Colombia Ecuador Mexico Panama Peru	Cross-sectional	1053	58.6	602	Convenience	Enteral nutrition Parenteral nutrition	SGA	Severe:233 Moderate: 512 Mild:261	NA
Velayati *et al*. (2019)	Iran	Cohort	398	60.9	306	Convenience	NA	BMI and SGA	Severe:22 Moderate: 162 Mild:214	17 month
Martins *et al*. (2017)	Brazil	Cross-sectional	328	61.4	182	Convenience	NA	Nutritional risk index	Severe:94 Moderate: 89 Mild:88	NA
Fischer *et al*. (2018)	Brazil	Cohort	66	64.1	39	Convenience	Oral	Nutritional risk index	Severe:3 Moderate: 2 Mild:8	90 days
Hachemi *et al*. (2015)	France	Cross-sectional	185	61.9	NA	NA	NA	SGA	Severe:23 Moderate:77	NA

**SGA: **Subjective Global Assessment, ** MUST: **Malnutrition Universal Screening Tool, **APMT**: Adductor pollicis muscle thickness, ** NUTRIC: **The Nutrition Risk in Critically ill, ** NRS-2002: **Nutrition Risk Screening 2002, ** MST: **Malnutrition Screening Tool, **BMI**: Body mass index, **CONUT**: Controlling Nutritional Status, **ABD-ASPEN**: Academy of Nutrition and Dietetics (Academy)/American Society for Parenteral and Enteral Nutrition

**Table 2:** Assessment of Study Quality Using the NOS

**Authors**	**Selection**	**Comparability**	**Exposure**	**Total**
Al-Kalaldeh *et al*. (2018)	3	1	2	6
Auiwattanakul *et al*. (2016)	3	1	1	5
Ceniccola *et al*. (2018)	4	1	1	6
Coltman *et al*. (2015)	4	1	3	8
Dos Santos *et al*. (2019)	1	0	2	3
Fetterplace *et al*. (2018)	2	1	2	5
Hiura *et al*. (2019)	3	0	1	4
Hope *et al*. (2017)	3	0	1	4
Kalaiselvan *et al*. (2017)	1	0	1	2
Kanekiyo *et al*. (2019)	2	1	1	4
Karst *et al*. (2015)	2	1	2	5
Lazarow *et al*. (2019)	2	0	1	3
Lew *et al*. (2018)	2	0	1	3
Lew *et al*. (2018)	2	0	1	3
Lew *et al*. (2019)	2	0	1	3
Marshall *et al*. (2017)	1	0	1	2
Rus *et al*. (2019)	3	0	1	4
Sharma *et al*. (2018)	3	2	2	7
Vallejo *et al*. (2017)	3	2	2	7
Velayati *et al*. (2019)	1	1	1	3
Martins *et al*. (2017)	2	2	1	5
Fischer *et al*. (2018)	3	0	2	5
Hachemi *et al*. (2015)	1	2	1	4
